# Prenatal diagnosis of meningomyelocele resolves as a mature cystic teratoma in the thoracolumbar region

**DOI:** 10.1007/s00381-024-06396-7

**Published:** 2024-04-29

**Authors:** Annie Chen-Carrington, Dean Leonard, Adam Goodreau, Jennifer Rhodes, Gary W. Tye

**Affiliations:** 1 Division of Plastic Surgery, Department of Surgery, West Hospital, Virginia Commonwealth University School of Medicine, 1200 East Broad Street, 16th Floor, West Wing, Box 980645, Richmond, VA 23298-0645 USA; 2 Division of Neurosurgery, Department of Surgery, West Hospital, Virginia Commonwealth University School of Medicine, 1200 East Broad Street, 16th Floor, West Wing, Box 980645, Richmond, VA 23298-0645 USA

**Keywords:** Chiari, Diastematomyelia, Meningomyelocele, Neural tube defect, Teratoma, Tethered spinal cord

## Abstract

A mature cystic teratoma is a mass with heterogeneous appearance, consisting of adult tissue with two or three layers: endoderm, mesoderm, and ectoderm. It is a rare, benign transformation of somatic tissue most commonly found in the sacrococcygeal region and may resemble an uncomplicated spina bifida on prenatal ultrasonography. In this case report, we describe a female newborn with an extremely rare mature cystic teratoma in the thoracolumbar region. She presented prenatally with a preliminary diagnosis of meningomyelocele, diastematomyelia, and Chiari II malformation and a possible teratoma. However, a mass containing solid glandular tissues and bony calcifications approximately 3 × 4 cm in size was observed in the thoracolumbar region upon birth. During surgical resection, no nerve roots were found in the associated meningocele. The patient retained full lower body function postoperatively following surgical excision of the thecal sac and teratoma.

## Introduction

**Fig. 1:**
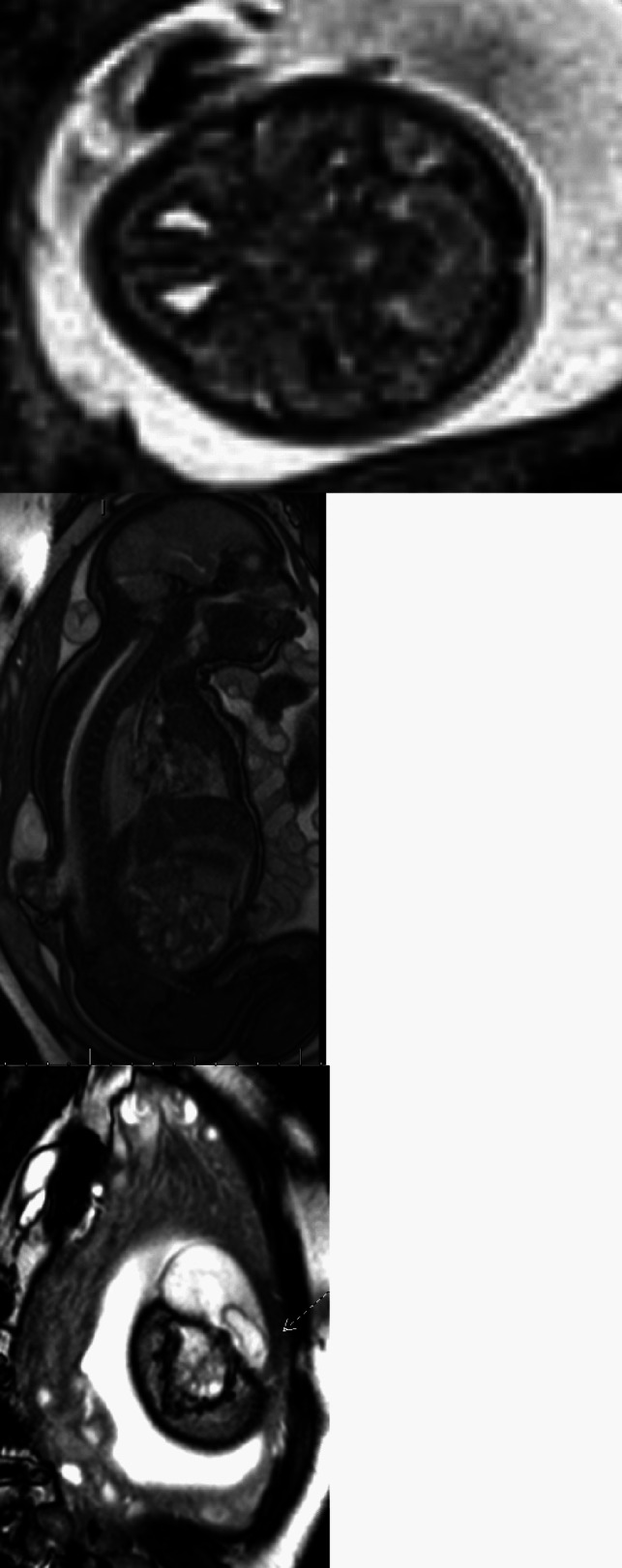
Prenatal ultrasonographic images showing lumbosacral neural tube defect

A mature cystic teratoma is a mass with heterogeneous appearance, consisting of adult tissue with two to three layers: endoderm, mesoderm, and ectoderm. Presentations in newborns with spinal teratomas are typically found in the sacrococcygeal area with a higher incidence in female infants (1 in every 35,000 live births) [1]. In this case report, we report a rare, mature cystic lumbar teratoma, which presented prenatally with a preliminary diagnosis of meningomyelocele, split cord malformation, Chiari II malformation and a possible teratoma. However, a thecal sac containing solid glandular tissues and bony calcifications approximately 3 × 4 cm in size was seen in the lumbar region after birth (see Fig. 1). Only one other case of this was reported by Balci et al. [2] in 2021 which discussed their experience with a mature cystic teratoma which also mimicked a meningomyelocele.

## Case report

**Fig. 2:**
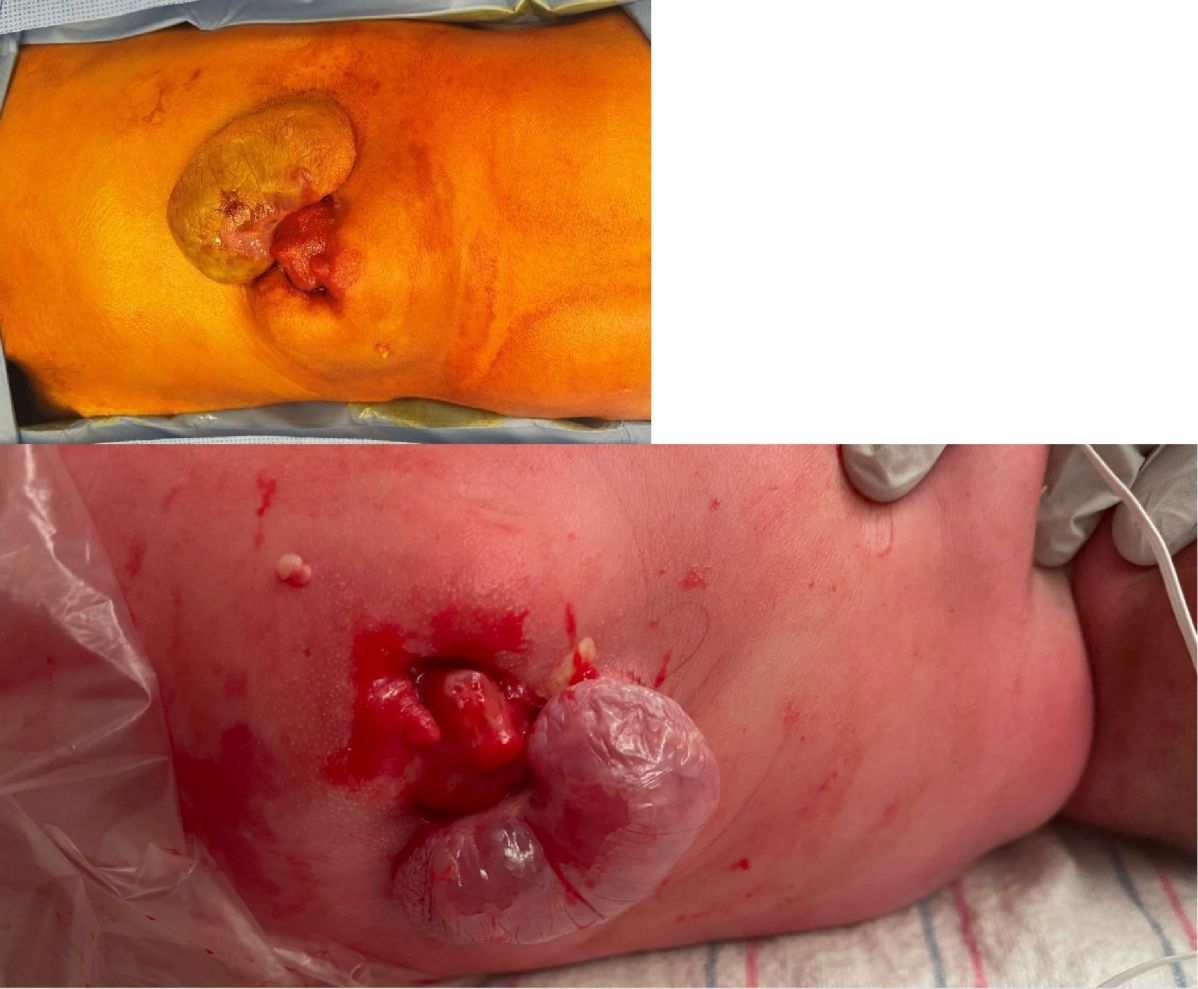
Postnatal evaluation of infant, a mass of 4 cm × 3 cm in the lumbar region, surrounded by a membrane containing both solid and cystic areas

**Fig. 3:**
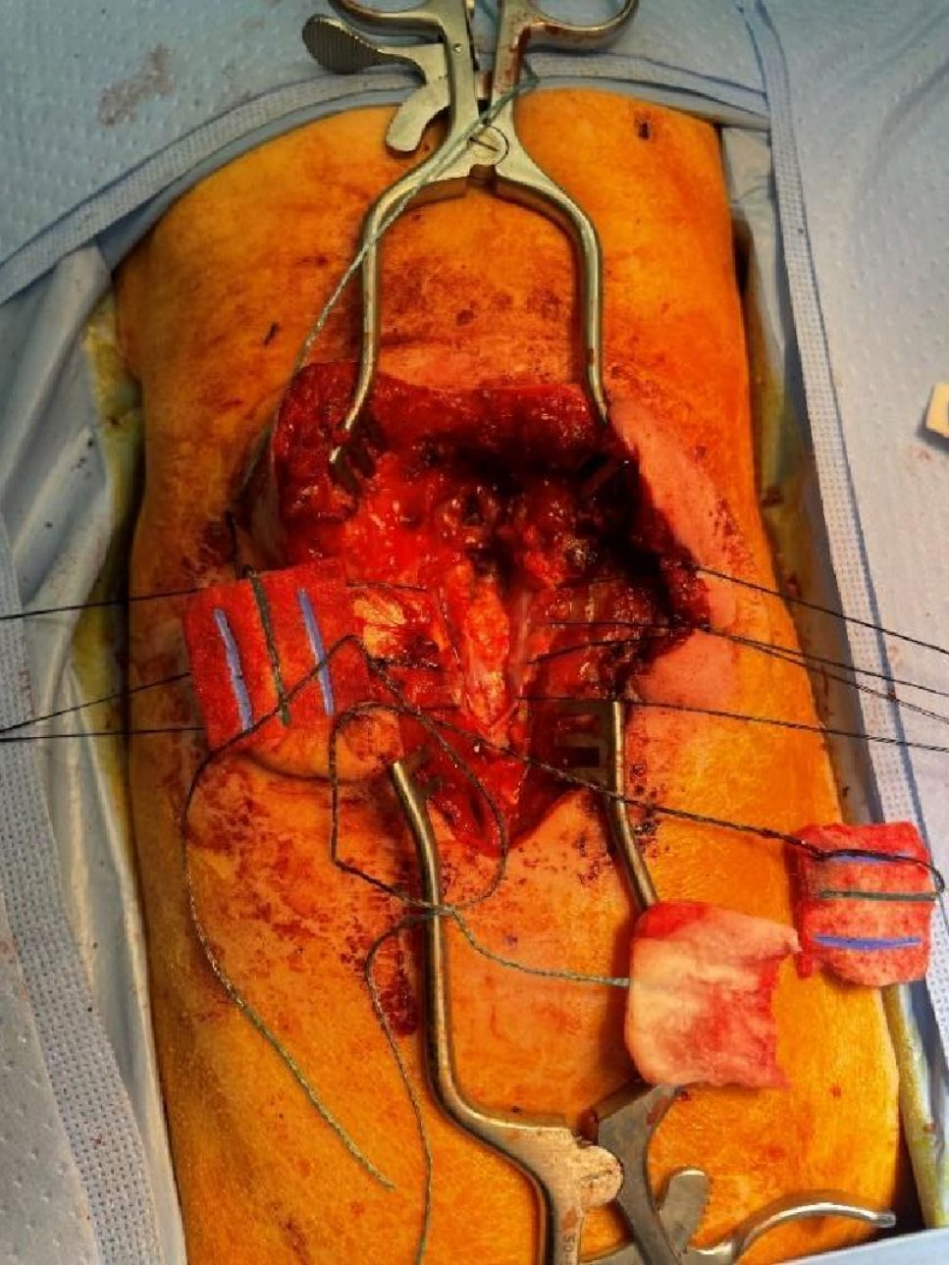
Following the mass excision, the dura mater was closed primarily and the paravertebral fascia was layered over it

**Fig. 4:**
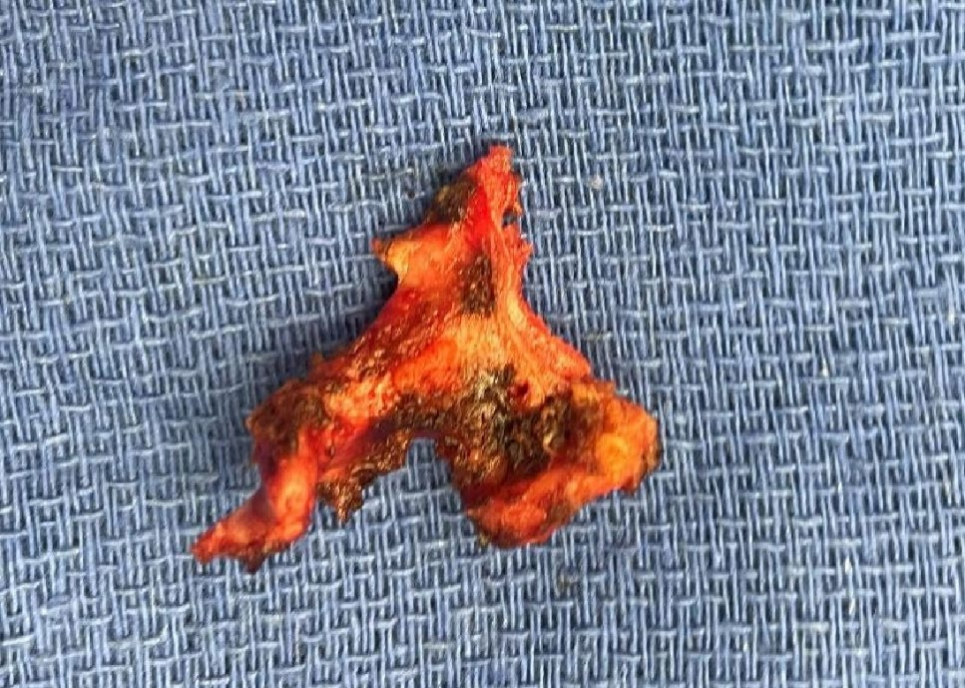
Solid part of mature cystic teratoma

**Fig. 5:**
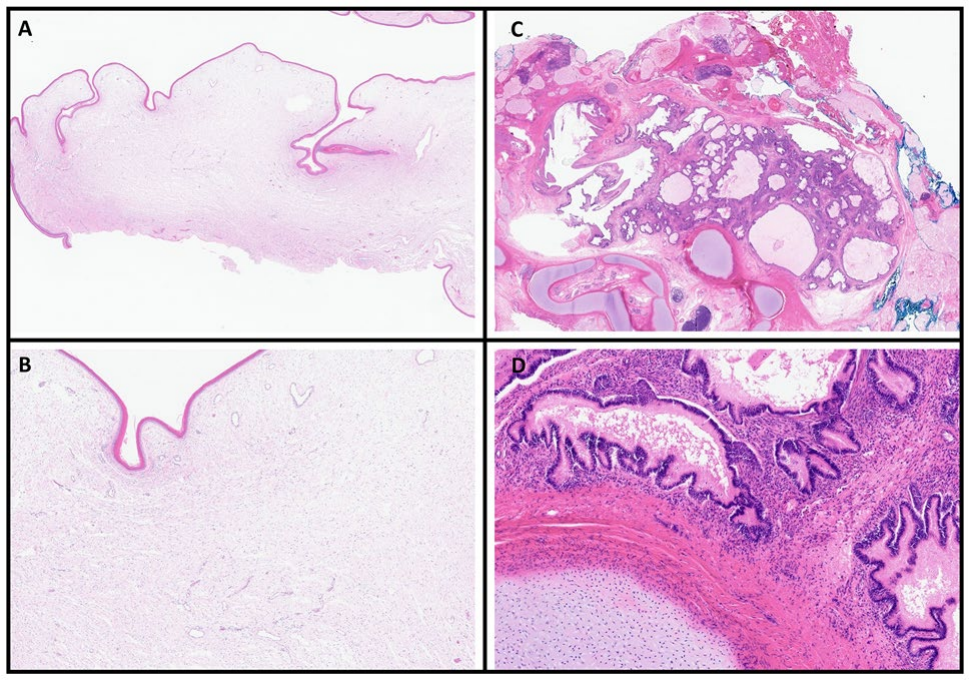
H&E stained sections of meningocele (**A** and **B**) and mature teratoma (**C** and **D**). **A** and **B** Skin with no appendages and underlying edema with dilated vessels. **C** and **D** Mass composed of mature cartilage, glandular tissue and adipose tissue

In this case study, our patient’s mother was a 42-year-old Caucasian female G2P1002, who presented with concern for spinal defects on routine prenatal ultrasound, but normal AFP levels. She had one child without incidence of previous spinal dysraphism anomalies. She took no relevant medications or had no relevant medical history to put her at higher risk for a child with a spinal dysraphism. She was sent for a 34-week fetal MRI as she was not a candidate for in utero repair. On the fetal MRI, the patient was found to have a lumbar midline defect. Following birth, a cystic sac containing solid glandular tissue and bony prominence, approximately 4 × 3 cm in size was seen in the thoracolumbar spine. On neurological examination, the female infant was moving her lower extremities proximally with hip flexion. Her feet were bilaterally flexed but there was spontaneous movement. A postnatal MRI demonstrated complex tethered cord pathology with multiple spina anomalies, including hemivertebrae and Chiari malformation. The patient underwent surgical repair on the day after birth under general anesthesia due to the presence of a thin covered meningocele and a concern for rupture (Figs. 2, 3, 4 and 5).

### Operative description

An incision above the lesion was made and the spinous process and lamina were then exposed in a subperiosteal fashion, and the laminectomy was carried out at the level just above where the soft tissue mass and the meningocele came together, at approximately the D12 level. This allowed for visualization of normal dura. Attention was then turned towards resection of soft tissue mass which was done by creating a plane above the thecal sac with mosquitoes. The soft tissue mass was then circumferentially exposed and removed from its underlying attachments where it came close to the intradural lipoma. The attention was then turned towards resection of the meningocele. On further careful exposure of the thecal sac, there was an attachment of the meningocele to the thecal sac and a silk tie was placed around the midline thecal sac with the adjacent attachment to the meningocele. At this point, the meningocele was entered using a 15-blade and bipolar electrocautery and tenotomies and no nerve roots were found within the meningocele. There was some fluid within the meningocele which was felt to be CSF due to direct communication with the thecal sac but was not further tested. It was determined that this was not a myelomeningocele and there were no neural components at the site of the silk suture, the meningocele was amputated. The attention was then turned towards resection of the intradural lipoma which had been visualized on the neuroimaging. To do this, the dura was opened in the midline using a 15-blade and a release of the tethered cord was then carried out using micro instruments. The tethering was caused by a lipoma, and the type 1 split cord malformation which were all tethered to it. The lipoma was on the dorsal aspect of the left portion of the cord above the split and at the right hemicord. The conus was at L5. During this portion, we were able to completely amputate the dorsal mass. It was attached dorsally to the lipoma OR Do You Mean Cord. No nerve roots were seen within the intradural lipoma. After doing this, the attention was turned towards the type I diastematomyelia and as expected, a bony septum was encountered at the level of the defect. There were several fibrous attachments dorsally to the split portions of the dura at the level of the split on the dorsal aspect at D11. These were carefully cut. The bony septum was then carefully exposed from all sides and removed with a combination of pituitary and bipolar electrocautery. Dura-Guard was then cut to size and 4–0 silk sutures were used to carry out a duraplasty. The wound was then closed by plastic surgery by bringing the paraspinal muscles together and closure was done in layers.

Post-operatively, the patient had normal neurologic function in the feet and voided well within the first day. She continues to develop appropriately and has not required a shunt.

## Pathology

Histopathological evaluation revealed that the resected meningocele sac was composed of skin with underlying dermis with edema and dilated vessels. There were no adnexal structures noted (Figures A and B) and the deep portion of the specimen showed a dense fibroconnective tissue that may represent dura. The mass-like lesion was composed of mature elements from all three germinal layers (Figures C and D). There was mature gastrointestinal epithelium, cartilage, adipose tissue, ganglion and cellular mesenchyme. No significant atypia or immature elements are present, consistent with a mature teratoma.

## Discussion

Teratomas are rare, mixed germ cell tumors that can occur in various locations, including the spinal region. Spinal teratomas, such as that in our case study, are a rare occurrence, estimated at 0.15–0.18% of all spinal cord tumors with a male-to-female ratio estimated at 3:1 [3]. Adult onset of spinal teratomas is rare and mostly observed in men during their 4th or 5th decades of life [4–7]. Spinal teratomas that emerge with concomitant spinal dysraphic lesions are presumed to be dysembryogenic in origin versus neoplastic [8]. There are a wide variety of theories developed to explain split cord malformations, though the most accepted is the unified theory by Pang et al. which proposes that the common committal step for all split cord malformations is the formation of an accessory neuroenteric canal, which results in the formation of an abnormal structure that results in the division of the notochord and neural plate [8, 9]. Balci et al. believe that the association of type I diastematomyelia and spinal teratoma may be related to the persistent neurenteric canal being used by pluripotent cells of the yolk sac, attempting to reach the gonads and instead become trapped [2].

The coexistence of a teratoma with other spinal anomalies and Chiari Type II malformation is an unusual presentation, with split cord malformation representing only 9.6% of concomitant congenital anomalies among those with spinal teratomas [10]. While an association between myelomeningocele and Chiari II is well established, the presence of meningocele and Chiari II is rarely reported in the literature. This discussion focuses on the relationship between spinal teratomas and spinal dysraphisms, of which there are limited studies in the literature.

Sharma et al. studied the incidence of spinal cord teratomas over 20 years by retrospectively analyzing their institution’s neuropathology records. In those 20 years, they found only 27 examples of spinal teratomas. Similar to other studies, their study found that there was a striking male predominance (4.4 males to 1 females) and that “77.7% of patients had concomitant dysraphic features in the form of anomalies of the vertebrae, split cord malformation, tethered cord and lipomeningomyelocele” [11, 12]. Their study recommends that spinal teratoma be a part of the pre-operative differential, with higher likelihood if imaging shows mixed solid and cystic morphology, fat signal, and areas of calcification. In addition, this study recommended surgical management of spinal teratomas as the treatment of choice, with radical removal of the tumor the aim of the surgical intervention whenever possible.

Babu et al. conducted a literature review of patients with split cord malformation with concurrent spinal teratoma. Their review found only eight cases and found that the lumbar spine was the most frequent location for teratomas (66.7%), with the Type II malformation more commonly occurring with these tumors (75%), with more than half occurring in females (55%) [13]. Babu et al. recommended the primary treatment of teratomas to be gross total resection due to possible progression of the remaining mass and potential impact on patient morbidity. During resection, authors recommended intraoperative electrophysiologic monitoring to prevent postoperative neurological deficits.

Another study by Hazneci et al. conducted a systemic review of literature for split cord malformation and concomitant spinal teratoma without any open spinal dysraphism. They found that spinal teratomas and split cord malformations “mostly presented at thoracic/thoracolumbar region in children and lumbar region in adults” with outcomes better in children than adults [14]. The study also found that split cord malformation was mostly diagnosed in pediatric cases in the thoracic or thoracolumbar regions. Hazneci et al. found that the treatment modality for pediatric patients who received surgical intervention was complete recovery, whereas only 27% of adult patients could manage to fully recover following surgeries, though this was not statistically significant. Of note, the treatment modality was unclear in 10% of patients, with outcomes unknown for 40% of patients in their study.

## Conclusion

Spinal teratomas and Chiari II malformation represent distinct yet complex challenges in spinal and neural anomalies. In cases where spinal teratomas are located in the lower spine, they may impact the dynamics of the neural tube closure, potentially contributing to the development of Chiari II malformation.

Our patient presented with an extradural thoracolumbar teratoma, intradural lipoma, type I diastematomyelia, meningocele, and a Chiari II malformation. The diagnosis of an extragonadal teratoma with Chiari II malformation was not able to be distinguished from meningomyelocele until surgical resection. Perinatal diagnosis, surgical excision, and accurate pathological diagnosis were essential to preventing future complications for the patient.

Since it is such a rare condition, carefully coordinated and multidisciplinary care was essential when addressing both conditions simultaneously. Both conditions require a comprehensive approach involving accurate diagnosis, tailored surgical interventions, and long-term monitoring for optimal patient outcomes. Further research and collaboration among medical specialties are crucial to advancing our understanding of spinal teratomas and Chiari II malformations and refining treatment strategies to improve the quality of life for affected individuals.
